# Advancing the Management of Long COVID by Integrating into Health Informatics Domain: Current and Future Perspectives

**DOI:** 10.3390/ijerph20196836

**Published:** 2023-09-26

**Authors:** Radha Ambalavanan, R Sterling Snead, Julia Marczika, Karina Kozinsky, Edris Aman

**Affiliations:** The Self Research Institute, Broken Arrow, OK 74011, USA; sterling@selfresearch.org (R.S.S.); julia@selfresearch.org (J.M.); karina@selfresearch.org (K.K.); edris@selfresearch.org (E.A.)

**Keywords:** COVID-19, post-COVID syndrome, concept map for management of long COVID, health informatics, public health, management of long COVID

## Abstract

The ongoing COVID-19 pandemic has profoundly affected millions of lives globally, with some individuals experiencing persistent symptoms even after recovering. Understanding and managing the long-term sequelae of COVID-19 is crucial for research, prevention, and control. To effectively monitor the health of those affected, maintaining up-to-date health records is essential, and digital health informatics apps for surveillance play a pivotal role. In this review, we overview the existing literature on identifying and characterizing long COVID manifestations through hierarchical classification based on Human Phenotype Ontology (HPO). We outline the aspects of the National COVID Cohort Collaborative (N3C) and Researching COVID to Enhance Recovery (RECOVER) initiative in artificial intelligence (AI) to identify long COVID. Through knowledge exploration, we present a concept map of clinical pathways for long COVID, which offers insights into the data required and explores innovative frameworks for health informatics apps for tackling the long-term effects of COVID-19. This study achieves two main objectives by comprehensively reviewing long COVID identification and characterization techniques, making it the first paper to explore incorporating long COVID as a variable risk factor within a digital health informatics application. By achieving these objectives, it provides valuable insights on long COVID’s challenges and impact on public health.

## 1. Introduction

**An epidemiological brief of COVID-19:** A cluster of pneumonia cases in Wuhan City, Hubei Province, China, with an unknown cause, raised concern among health officials in late December 2019. On 31 December 2019, a forewarning was issued by the Wuhan Municipal Health Commission, an instantaneous response team was directly sent to Wuhan by the Chinese Center for Disease Control and Prevention (China CDC), and a notification was made to the World Health Organization (WHO). Genomic characterization and testing methods were developed after identifying the causative pathogen as a novel coronavirus on 7 January 2020. On 30 January 2020, WHO declared the emergence of the novel coronavirus (2019-nCoV) a public health emergency of international concern (PHEIC) and subsequently named it severe acute respiratory syndrome coronavirus 2 (SARS-CoV-2) and the disease Coronavirus Disease-2019 (COVID-19) on 11 February 2020. On 11 March 2020, WHO announced COVID-19 as a pandemic. Six events were declared PHEIC between 2007 and 2020: the H1N1 influenza pandemic (2009), poliomyelitis (2014 to present), Ebola in the West African outbreak (2013–2015) and the outbreak in the Democratic Republic of Congo (2018–2020), and Zika (2016) [1,2,3,4].

COVID-19 spreads mainly by droplets being produced from the coughing or sneezing of a COVID-19-infected person, as well as contaminated surfaces, the nose, mouth, or face. COVID-19 screening includes the analysis of samples to evaluate the current (molecular tests) or past presence of SARS-CoV-2 (antibody tests) [5]. The symptoms may appear 2–14 days after exposure to the virus, and include fever, shortness of breath, cough, sore throat, chills, congestion or runny nose, headache, fatigue, muscle pain, and the loss of taste or smell. Other less common symptoms include gastrointestinal symptoms such as nausea, vomiting, or diarrhea. It should be noted that not all individuals infected with COVID-19 manifest all the associated symptoms, while a subset of individuals may remain asymptomatic throughout the course of the illness [6]. Although age is the most critical risk factor for the severe consequences of COVID-19, patients with some underlying health conditions are more susceptible to an increased risk of unfavorable outcomes and higher mortality rates [7]. Acute COVID-19 infection is less common and less severe in children, as has been demonstrated repeatedly by scientific studies [8]. However, as with the entire global population, children have been affected by lockdowns, limited schooling, and social interactions, which may have indirect and permanent mental and physical health implications. COVID-19 containment is through pharmaceutical (i.e., vaccination) and non-pharmaceutical interventions (e.g., mask use, physical distancing, hand hygiene, isolation, and quarantine) [9,10].

The COVID-19 pandemic has been sustained by the emergence of new virus variants [11]. Throughout the waves of the pandemic, the world has experienced different virus variants, including the alpha, beta, gamma, delta, and omicron variants. These variants have been classified by the World Health Organization (WHO) using Greek alphabet letters based on their specific characteristics as either variants of concern (VOCs) or variants of interest (VOIs). Despite efforts to achieve herd immunity through global vaccination campaigns, new variants continue to appear, accompanied by a resurgence in cases, posing challenges in understanding the intricacies of SARS-CoV-2 infection [12,13]. Additionally, these variants have had various impacts on disease diagnosis, treatment, vaccination effectiveness, and long-term health consequences following SARS-CoV-2 infection.

Two years after the declaration of the pandemic, SARS-CoV-2 continues to pose a global burden with a severe threat to health and wellness and global stability. The situation remains challenging, with limited options for managing patients’ infection control and severe disease outcomes even in countries with an advanced hospital infrastructure. WHO reported more than 750 million confirmed COVID-19 cases and more than 6.5 million related deaths as of 10 June 2023 [14,15].

While daily reported cases of mortality and morbidity have gone down significantly due to notable successes in the development and distribution of vaccines, the urgency to comprehend and address the impacts of long COVID remains high [16,17]. Although our understanding of this condition remains limited, a number of individuals who have recovered from COVID-19 have reported enduring or new symptoms, including shortness of breath, cognitive difficulties, persistent fatigue, and depression. These symptoms have the potential to incapacitate millions of people worldwide and can persist for weeks, months, or even years, significantly affecting the daily lives of a substantial portion of recovered patients [18,19]. There is a concern that this phenomenon could potentially give rise to a second public health issue, adding to the existing challenges posed by the pandemic. To effectively devise strategies for the prevention, treatment, and support of individuals with long COVID, it is essential to acquire a comprehensive understanding of its origins and impacts, as well as related conditions. This necessitates research into the underlying biological mechanisms, the identification of effective treatment approaches, and the establishment of necessary services and support systems for those affected by long COVID. We intend to include the new unifying essential frameworks that have been included for long COVID as a risk factor variable in the digital health informatics app. In addition to laboratory tests, a range of management strategies are listed for identifying and characterizing long COVID and advancing the understanding of the long COVID syndrome by AI, particularly identifying individuals with long COVID using a machine learning (ML) approach.

In this study, we aimed to bridge scientific gaps in long COVID management by creating a comprehensive concept map at a higher level rather than specific details. To achieve this, we conducted an extensive literature review to identify the existing knowledge gaps and explore various possibilities for holistic health interventions. The first phase of our study involved an in-depth literature review conducted from September 2020 to October 2022, using the PubMed databases and specific search terms, including “long COVID”, “long COVID characterization techniques”, “long COVID in health informatics”, and “public health impact by long COVID”. Boolean operators (AND and OR) were used to find articles containing variations of the specified search terms. Fourteen studies were included in the review, out of which eight were retrospective studies. The inclusion criteria involved articles written in English and published in peer-reviewed journals to ensure the quality of the research. This study contributes to the ongoing efforts to effectively manage the long-term effects of COVID-19 and improve public health preparedness for future infectious disease outbreaks by establishing a solid foundation for further research and development. We plan to develop a strong research roadmap to guide prospective studies in this field based on the comprehensive knowledge gained from this review paper on managing long COVID.

## 2. Long COVID: A Concise Contextual Future

**An interim definition of long COVID:** International patient organizations founded almost immediately after the first SARS-CoV-2 cases have continued to raise awareness of the potential long-term consequences of acute illness [20]. In some instances, patients with asymptomatic illness describe symptoms that develop weeks or even months after the first phase of the infection has gone. The term “long COVID” is used to describe these collections of persistent symptoms that occur after the initial COVID-19 infection. This group of conditions is also referred to as “long haulers”, “post-acute COVID-19 syndrome (PACS)”, “post-acute sequelae of SARS-CoV-2 (PASC)”, “post COVID-19 condition (PCC)”, “post COVID syndrome”, “long-term COVID”, and “chronic COVID.” These conditions encompass a broad range of long-lasting or newly emerging symptoms that persist for more than 12 weeks and, in some cases, up to 2 years so far [21,22].

**Figure 1:**
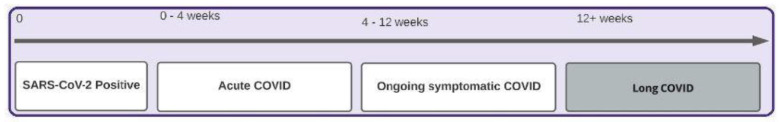
Timeline for acute and long COVID.

On 30 October 2020, the National Institute for Health and Care Excellence (NICE), the Scottish Intercollegiate Guidelines Network (SIGN), and the Royal College of General Practitioners (RCGP) jointly proposed an integrative definition of long COVID as symptoms that persist or develop after acute COVID-19. The following long COVID classification has been defined as (i) acute COVID-19 (symptoms for up to 4 weeks), (ii) ongoing symptomatic COVID-19 (symptoms for 4–12 weeks), and (iii) post-COVID-19 syndrome (symptoms formed during or after infection and continuing for more than 12 weeks) (Figure 1). The term “long COVID” in this guideline refers to both subgroups, ongoing symptomatic COVID and post-COVID syndrome [23,24]. In October 2021, the World Health Organization applied the Delphi consensus technique to establish a unified clinical case definition, using the phrase “post-COVID-19 condition” [22].

**Pathophysiology:** The underlying cause of long COVID is still not fully understood, and researchers have proposed various hypotheses to explain its pathophysiology. It is believed that long COVID may arise through multiple mechanisms, including the persistence of the virus in reservoirs such as the epithelial tissue in the small intestine, which allows the virus to survive and persist in the body [25]. Other potential mechanisms include autoimmune responses, activation of the immune system mediated by SARS-CoV-2 superantigens [26], hyperactivation of blood coagulation and platelets, as well as direct effects of viral infection and subsequent inflammatory or autoimmune reactions [27]. Long COVID may manifest as the emergence of chronic health issues, encompassing a range of conditions such as heart disease, diabetes, kidney disease, hematologic disorders, and mental and neurological illnesses. It is a complex condition that can affect almost every organ system in the body.

**Diagnosis:** WHO, in consultation with stakeholders in International Classification of Diseases (ICD) codes, specifically ICD-10 and ICD-11 [28,29], introduced a new ICD-10-CM diagnosis code, U07.1, for confirmed cases of acute SARS-CoV-2 infection, and code U07.1/2 was designated for suspected cases in January 2021. In the updated 2023 version of ICD-10-CM, a different diagnosis code, U09.9 (“Post-COVID-19 condition”), was explicitly assigned for long COVID [30,31]. Healthcare systems need to adopt this code for standardized reporting and improved monitoring. Despite the progress in recognizing long COVID as a specific diagnosis, several challenges persist in clinical practice. For instance, not all practicing doctors are currently familiar with the diagnosis of long COVID. More complex clinical diagnostics are needed to detect the condition accurately.

**Lingering Signs and Symptoms:** The signs, symptoms, and conditions continue or reappear four weeks or more after the initial symptomatic or asymptomatic infection; they may also be relapsing and remitting, with repeated fever spikes being a prevalent feature [26,32]. It may be singular, numerous, stable, transient, or fluctuating, and can vary over time. The most prevalent signs and symptoms may relate to multiple organ systems such as respiratory, cardiovascular, gastrointestinal, neurological, psychological/psychiatric, musculoskeletal, dermatological, endocrine/genitourinary, and post-traumatic stress disorder (PTSD) [33]. However, clinical manifestations might fluctuate. In fact, more than two hundred biomedical findings have been recorded, with many patients exhibiting dozens of symptoms spanning many organ systems. At least 25% of afflicted individuals describe symptoms, such as discomfort, that interfere with their everyday activities [34,35]. The possibility of the potential clustering of these symptoms or the risk factors that may predispose some people to experience specific clusters is still unknown, as there is uncertainty regarding diagnosis, treatment options, and population-level risk factors [36]. As a result, long COVID is frequently described as a single syndrome. However, in view of the heterogeneity of long COVID observed so far, there is a strong indication that it is probably not a single condition but rather a combination of several conditions. With an increasing number of individuals reporting a wide range of persistent symptoms, a one-size-fits-all treatment plan is not viable. Moreover, the comprehensive understanding of the underlying physiological mechanisms, the long-term prognosis, and the optimal therapeutic interventions for long COVID may be hampered if treated as a single condition [37,38].

A rigorous scientific investigation and hazard mitigation effort is needed to proactively identify this heterogeneous condition’s characteristics, especially given the evolving nature of the symptoms experienced by long COVID patients. It is imperative to record the entire spectrum of symptoms experienced by long COVID patients to increase our knowledge of their underlying pathophysiology and disease process, as well as for characterizing associated phenotypes and risk factors, thereby improving the efficacy of clinical trials and advancing treatment options.

Moreover, the epidemiology of long COVID, its prevalence, is unclear. These epidemiological statistics are vitally needed for long COVID care planning to prevent medical system overload or the creation of inappropriate or excess care facilities. However, obtaining these kinds of data via clinical studies might take time due to the vast prevalence ranges indicated in the available evidence.

### 2.1. A Phenomenal Burden of Long COVID

The increasing number of COVID-19 cases has led to a rise in individuals experiencing prolonged symptoms, causing challenges to patients, their families, and the economy, and potentially impacting worldwide workforce productivity and healthcare systems. Adequate resources, research, and support services are necessary to address the unique needs of long COVID patients and alleviate the strain on individuals and healthcare systems.

Along with 13 other federal agencies, the Department of Health and Human Services (HHS) Office of the Secretary [39], the Centers for Disease Control and Prevention (CDC) [40], and the National Institutes of Health (NIH) [41] developed interim definitions of long COVID and contributed to the research plan with input from patients, academics, and other stakeholders. The medical, scientific, and public health communities including WHO have developed precise terms for communicating with agreed-upon, interim definitions of long COVID such as long haulers, chronic COVID, PCC, PACS, and PASC [22,32]. It is essential to properly separate the different long COVID conditions using standard names, which would benefit from further investigation and global surveillance. Interdisciplinary monitoring is required for all patients to identify long COVID symptoms before long-term systemic impact occurs.

Global Burden of Disease Long COVID Collaborators [42] conducted an observational analysis to ascertain the prevalence of symptoms of long COVID in patients three months after symptomatic COVID-19. They conducted a comprehensive review of 54 studies and two medical record databases spanning 22 countries. Data from approximately 1.2 million individuals were analyzed to estimate the global proportion of individuals who continued to experience symptoms of long COVID after symptomatic COVID-19 infection between 2020 and 2021. The findings revealed that 3.7% of individuals had persistent respiratory symptoms, 3.2% reported enduring fatigue accompanied by bodily pain or mood swings, and 2.2% experienced cognitive problems three months after the initial infection. Moreover, 6.2% of patients exhibited any of the aforementioned symptom clusters. Notably, at the 12-month mark, 15.1% of individuals who had experienced long COVID symptoms for more than three months still reported ongoing symptoms. According to the CDC’s epidemiological team, Bull-Otterson et al. [43] assessed electronic health records (EHR) data from March 2020 to November 2021 and found that one in five (20%) adult COVID-19 survivors aged 18 to 65 and one in four (25%) survivors aged 65 and above experienced at least one incident condition that might be attributable to their prior COVID-19 illness, despite estimates of the overall risk of long COVID ranging between 5 and 30%. According to another mathematical modeling research conducted by the CDC, it has been estimated that a significant number of Americans experience long-lasting symptoms that restrict their daily activities after contracting the COVID-19 virus. The study also suggests that women may be disproportionately affected by these symptoms [44].

Authentic information on the number of global sufferers living with long COVID is unclear. The UK Office for National Statistics (ONS) estimated that as of November 2022, 2.1 million people in the UK (3.3% of the population) had indicated the presence of self-reported long COVID symptoms lasting more than four weeks (point in time prevalence estimate) [45]. Although there may be variations in estimates regarding the burden of long COVID across different studies and settings, the range of symptoms and diseases that are manifestations of long COVID and the potential scale remain constant in the scientific literature. Long COVID is real, affecting a significant number of people, and could continue to rise as new infections occur.

Health system planning and resource allocation relies on reliable estimates of disease burden, but a lack of high-quality data inhibits these efforts. It is worth noting that individuals who have previously tested positive for COVID-19 have been observed to utilize medical services more frequently [46]. It would be beneficial if public health officials could implement proactive measures to mitigate the risks associated with long COVID, at both the community and population level [47]. Integrated healthcare settings are essential for facilitating communication between providers regarding patient healthcare plans and progress and avoiding pointless repeat tests, which will save patients’ time and medical expenses. Policy and practices also need to be established to effectively harness and optimize collaboration between medical specialties, multiple healthcare professionals, and patients.

#### 2.1.1. Outlining the Hurdles in the Field of Economics

Ongoing research on long COVID suggests it has considerable adverse effects on society and the economy. Preliminary estimates indicate that it could result in approximately 1 million full-time equivalent workers being unable to return to work in the US, causing a loss of around USD 170 billion in wages, equivalent to nearly 1% of the country’s gross domestic product [48,49]. According to a comprehensive analysis of the available research and existing data, the incidence of long COVID is estimated to be 10–30% among non-hospitalized cases, 50–70% among hospitalized patients [50,51,52], and 10–12% among vaccinated individuals, with significant repercussions for the health system and economy [53]. The economic repercussions of the epidemic will take years to properly assess, but they are massive. Considering the entire economy, long COVID may result in over one million individuals being unemployed at any given moment [48].

Following the acute phase, the management of persistent COVID-19 symptoms and associated complications results in heightened healthcare utilization and financial strain for both patients and the healthcare system [54]. The economic implications of long-term COVID care structures manifest through concerns about resource capacities. The scarcity of specialized rehabilitation centers and restricted access contribute to long waiting lists, while patient preferences and geographical constraints can further exacerbate the issue. The emergence of private providers offering expedited services accentuates the existing two-tier healthcare system [55].

The rising number of long COVID cases and the corresponding rising average sick leave durations may pose potential strains on the economy, particularly the labor force. Since COVID-19 and the long COVID association have impacted a substantial percentage of the population, the future medical requirements for people and the costs associated with them are inadequately understood, which may result in patients enduring prolonged financial strain. The policymaker must be aware of the repercussions for the healthcare system to develop well-designed policy planning for the post-pandemic world. A future review of the financial sustainability and total cost of providing care for persons with long COVID is required. Added to this, variants of SARS-CoV-2 have made it harder for individuals to stay healthy and more difficult for vaccines to protect them. In the pretext of the continued presence of the virus, it is crucial to acknowledge that individuals may live with it, indicating that COVID could potentially remain a significant threat to both the economy and healthcare.

#### 2.1.2. Exploring the Societal Impact of Long COVID

Rehabilitation specialists possess the necessary expertise to address the complexity of post-acute functioning owing to their extensive training in managing debilitating conditions while prioritizing the enhancement of quality of life. However, some patients may worry about reintegrating into society after completing an inpatient rehabilitation program. Accordingly, it is suggested to devise specific measures of follow-up, such as a practice plan for continuing exercises at home, a referral to outpatient non-medical healthcare treatments, or the provision of social worker assistance [56,57]. Moreover, the CDC has issued a set of health equity guiding principles for inclusive communication [58], which underscore the need for inclusive and respectful communication with all populations.

According to these perspectives, effective communication between healthcare practitioners and patients entails the provision of health resources in simple language and in the languages spoken by patients, imparting training to healthcare professionals on the best practices, and reviewing health materials such as insurance forms and medication instructions with community members to ascertain their comprehension of the information. Therefore, achieving an appropriate equilibrium at the information level is crucial to mitigate unnecessary uncertainty.

Sustained health consequences may necessitate a range of resources for recovery, including clinical treatments such as rehabilitation and mental health services, in addition to social support and other resources to facilitate the maintenance of responsibilities and social engagement. It is imperative to analyze general practitioner availability considering the anticipated increase in long COVID patients and their significant role in therapy. It is worth considering whether non-medical healthcare practitioners, such as physiotherapists or psychotherapists, should be accessible to patients as an initial point of contact. Doing so could reduce the burden on primary care doctors.

#### 2.1.3. The Changes in Quality of Life as an Outcome of Long COVID

The state of one’s health plays a vital role in determining a person’s capacity to lead a satisfying life. It is important to note that chronic diseases can have a significant impact on a patient’s overall health, potentially limiting their ability to live well and affecting their functional status, productivity, and health-related quality of life (HRQoL) [59,60]. One potential approach to assess the well-being of individuals experiencing long COVID is to utilize various standardized tools, such as the 5-level EQ-5D version (EQ-5D-5L) index score [61], EuroQol Visual Analog Scale (EQ-VAS), RAND 36-Item Short Form Health Survey (SF-36) [62], the 12-Item World Health Organization Disability Assessment Schedule (WHODAS) 2.0., the 10-Item Patient Reported Outcome Measurement Information System (PROMIS) Global Health [63], and the Post-COVID-19 Functional Status Scale (PCFSS) questionnaires [64]. Based on current evidence, individuals with long COVID are likely to experience a substantial decline in their overall quality of life. Moens et al. [65] conducted a study to ascertain the HRQoL of patients who had been experiencing long COVID symptoms after a mild to moderate SARS-CoV-2 infection. The research compared the HRQoL between patients from prepandemic and pandemic cohorts. The EQ-5D-5L can be used to estimate HRQoL scores. The results suggest that prolonged manifestations of COVID-19 significantly impact the patient’s quality of life, leading to an overall escalated situation in the disease’s burden. However, it remains unclear how patients with long COVID syndrome, who had a mild or moderate acute infection, are affected in terms of HRQoL.

The persistence of health issues that affect living conditions, social support, employment status, and financial security can have an adverse impact on an individual’s quality of life. Additionally, it would be advantageous for policymakers to consider the increasing population of individuals who are dealing with multiple chronic illnesses as well as those who are experiencing long-term effects of COVID-19, and to consider how their lifestyles may be affected. By recognizing the unique challenges faced by these individuals and implementing appropriate policies, governments may provide better support and resources to enhance their quality of life and promote overall well-being.

### 2.2. Accelerated Research and Path-Breaking Identification of Long COVID: A Brief Timeline

After the initial pandemic outbreak in April 2020, anecdotal patient reports indicated that previously healthy people were still exhibiting symptoms and had not entirely recovered from SARS-CoV-2 infection. In May 2020, a decentralized group of individuals who had been affected by COVID-19 released a survey about the various symptoms experienced by people with long COVID [66]. A detailed systematic characterization of the condition was provided by large-scale controlled reports conducted by Al-Aly et al. [67] early in 2021 using administrative data from the Veterans Affairs health system in the United States. These findings also supported widespread symptoms impacting many organ systems, including neuropsychiatric, respiratory, metabolic, cardiovascular, and gastrointestinal diagnoses. In the middle of 2021, Davis et al. [34] released studies documenting the range of organ failure in long COVID patients. Moreover, in line with the research, 22% of those with long COVID were unable to work owing to health reasons, while another 45% had to limit their hours due to health concerns. Between March 2020 and October 2021, when the original virus and its Alpha and Delta variants were circulating, researchers in Israel conducted a nationwide cohort study to assess the outcomes of about 300,000 infected patients. Except for certain cases such as reduced dyspnoea in vaccinated patients, the risks associated with most long COVID symptoms were found to be similar between unvaccinated or vaccinated people [68]. Children had fewer outcomes than adults, and most symptoms or conditions that developed after mild COVID-19 infection lingered for several months but returned to normal within a year. Researchers from the International Severe Acute Respiratory and Emerging Infection Consortium (ISARIC) pediatric long COVID working group in the UK, Sechenov University, and the Z.A. Bashlyaeva Children’s Municipal Clinical Hospital in Russia conducted the largest study on COVID-19-hospitalized children [69]. At the follow-up, there was a higher chance that the symptoms would still be there if the person was older or had an allergy disease. Parents said they were tired, had changes in taste or smell, and had trouble sleeping, with insomnia being the most common problem. In early 2022, Xie et al. [70] recorded the significant burden of long COVID; it is a non-monolithic disease with consequences that vary in expression between populations. A wide variety of clinical phenotypes have been identified, encompassing clusters of symptoms, discrete clinical episodes, organ-specific presentations, and multisystem syndromes [71]. In the middle of 2022, Thompson et al. [72] conducted a study of a large sample of patients seeking care in the United Kingdom that indicated pre-existing medical and mental disorders, obesity, and asthma are significant risk factors for long COVID. Additionally, demographic variables such as older age, female sex, and race were found to be important factors. In late 2022, Chen et al. [73] reported a meta-analysis and systematic review of the global prevalence of long COVID. According to their estimation, up to 43% of COVID-19 survivors worldwide reported persisting symptoms four months after diagnosis. The researchers hypothesize that the Omicron variants may impact the prevalence of long COVID and symptom burden in the future. They also highlight that it is challenging to synthesize research due to various standards for long COVID diagnoses worldwide. According to a worldwide meta-analysis report of 194 research studies by O’Mahoney et al. [74], the pooled prevalence of long COVID across hospitalized and non-hospitalized patients was around 45%.

In early 2023, Wang et al. [75] found that leading a healthy lifestyle reduces the incidence of long-term COVID in a dose-dependent way. In women, a correlation between five to six pre-infection healthy lifestyle factors had a 49% lower risk for long COVID compared with women participants who had none of those lifestyle factors. According to a new Kaiser Family Foundation analysis of the Household Pulse Survey [76,77] taken on 26 January 2023, the percentage of respondents who had COVID-19 during the pandemic and are reporting long COVID symptoms decreased from 19% in June 2022 to 11% in January 2023.

Scientists aim to enhance their comprehension of long COVID by examining its prevalence, duration, and clinical outcomes. Extracting essential information from longitudinal data, which encompass symptoms, laboratory results, images, functional tests, genomics, mobile health/wearable devices, written notes, electronic health records (EHR), and other relevant data types, presents a complex challenge. This complexity arises due to the substantial volume and detailed nature of the data generated during contemporary healthcare interactions. As a result, advanced analytical methodologies are required to effectively tackle this task. AI and, more specifically, ML techniques have increasingly demonstrated the potential to provide insights into patient-specific data obtained from extensive amounts of information, thereby improving our comprehension of the effects of SARS-CoV-2 on patients. This phenomenon can be attributed to the advanced sophistication of software tools and increased computing capabilities [78].

### 2.3. Long COVID Research—A Strategy and Action Plans to Document the Complex Variables

Anyone, including children, is at risk of having long COVID; it can appear in those who previously had asymptomatic, mild, or severe cases of COVID-19. Because there is little evidence to guide physicians on treatment, it is critical to identify the many endotypes that underpin the disease. More evidence and studies from multidisciplinary teams are required to fully understand the causes, mechanisms, and risks. The clinical manifestations and associated conditions of long COVID may vary with age, demographics, viral mutations, reinfections, and interventions such as vaccines; thus, it is vital to research topics to explore the risk factors. Further knowledge of these consequences is required to build specific, dynamic cross-sectoral therapies.

**Significance of All the Existing Evidence:** To continue providing vital healthcare services while combating the COVID-19 pandemic, meticulous planning and concerted action are required. Surveillance systems are very effective tools when it comes to tracking and sharing information about various health events, such as infectious diseases, chronic diseases, congenital disabilities, environmental and occupational health, and injury control [79]. Public health surveillance data are collected from a wide variety of sources. Each source of information provides a different overview of the frequency and distribution of disease; incorporating information from multiple sources can help to build up a more complete and accurate picture [80]. The COVID-19 pandemic has spotlighted infectious disease surveillance systems and the importance of making such data widely accessible. Sharing surveillance data on time can help a wide range of public health research projects, such as predicting the spread of illness, simulating potential therapies, and keeping the public informed about outbreaks [81]. Considering these values, several groups have spent the past two years attempting to increase access to large-scale epidemiological datasets [82]. The most recent COVID-19 efforts include the WHO-HQ COVID-19 surveillance database [83], the U.S. NIH’s N3C [84], the Datavant COVID-19 research database [85], the CDC’s COVID-19 case surveillance databases [86,87], and the Global health data science initiative [88]. Beyond COVID-19 infection surveillance, healthcare organizations must merge long COVID to monitor patient burden, recovery, and progression.

Massive development has occurred in recent years in the use of EHR and the quantity of stored digital health data. COVID-19 revealed the uses of digital technology in healthcare. Digital technology may help reduce virus transmission by providing medical advice, administering treatment, and tracking viral spread. During the COVID-19 pandemic, epidemiological data about viral sequences and human demographics have been collected worldwide. Biomedical research has become data-intensive in the era of information technology and big data, with the development of increasingly vast, complex, multidimensional, and heterogeneous datasets. A vast amount of open-source software is available, such as the Apache project’s big data components, that are intended to work in a cloud computing and distributed environment to aid in creating big-data-based solutions. Furthermore, big data have numerous crucial qualities known as the six V’s: value, volume, velocity, variety, veracity, and variability, deriving more value and becoming more patient-centric [89,90].

In response to the COVID-19 pandemic, the US informatics and clinical communities analyzed an enormous amount of EHR data to identify possible risk factors and COVID-19 therapeutics. A national clinical database program, NIH’s N3C [91] and RECOVER [92,93], was launched to obtain information on long COVID consequences. WHO has created a Global Clinical Platform [94] and standardized case report forms (CRF) for acute COVID-19 and post-COVID-19 conditions (post-COVID-19 CRF) that are available to all member states and interested parties to facilitate the systematic gathering of similar data throughout the globe. For COVID-19 research, the N3C provides one of the biggest repositories of secure, de-identified clinical data in the United States, and it is now the most comprehensive publicly accessible Health Insurance Portability and Accountability Act (HIPAA). The N3C Data Enclave is gathering clinical data from population-based longitudinal studies (LS) on long COVID, including a variety of information from EHR data across multiple clinical organizations in the United States, including the Clinical and Translational Science Awards (CTSA) Program hubs, laboratory findings, imaging results, functional studies, genomics, health monitoring via wearable devices, demographic data, information on social determinants of health (SDOH), and others. The Observational Medical Outcomes Partnership (OMOP) common data model, version 5.3.1, is used by N3C to synchronize EHR data across four clinical data models and to provide a single analytical platform. N3C’s longitudinal data from medical records for patients with COVID-19 provide a robust strategy for developing machine learning algorithms to identify characteristics of patients who may have long COVID and variables that may help identify such patients. The research aims to discover long COVID subtypes to make the disease simpler to study and cure [95].

In February 2021, the RECOVER program launched an initiative with a budget of over USD 1B to support research on long COVID to improve understanding of long COVID and to declare the outcome of safe and effective diagnostic, treatment, and preventive strategies. It includes longitudinal observational clinical cohort studies with hundreds of diverse participants throughout their lifetimes. The RECOVER program will also perform clinical trials [96] on the efficacy of promising medicines to decrease the symptom burden in subgroups of people with long COVID [18,92,97].

To supplement the NIH’s existing long COVID research programs, such as RECOVER, the Rapid Acceleration of Diagnostics (RADx)-Radical (RADx-rad) program at the NIH is introducing the Long COVID Computational Challenge (L3C). The challenge is intended to encourage innovative, data-driven solutions that contribute to a better understanding of the risks of developing long COVID. The primary goal is to create open-source tools, such as AI/ML models and algorithms, that can be used with structured medical data to determine whether patients have a high risk of acquiring long COVID [98]. In April 2022, the U.S. Department of Health and Human Services [99] released a National Research Action Plan [100] on long COVID to encourage public and private researchers to accelerate their research.

### 2.4. The Novel Health Informatics Approaches to Understanding Long COVID Clinical Variables

In the 1970s, advances in AI and the development of information-based frameworks led to the need to automate the process of creating and maintaining knowledge bases. The proliferation of AI drove this requirement. Due to technical advancements, AI in modern medicine is a burgeoning domain in detection, grading, genome analysis, diagnostic imaging, including image analysis, decision making, and prognosis prediction, characterizing diseases, and control measures [101,102]. In the field of AI, the phrases deep learning (DL) and ML are of paramount significance. The field of ML involves the utilization of algorithms to enable computers to learn from data and execute tasks without explicit programming. On the other hand, DL employs intricate algorithms that are designed to mimic the structure of the human brain, enabling the processing of unstructured data such as text, images, and documents. DL is a distinct subfield of ML, which in turn is a subcategory of AI [103,104].

In order to provide the best possible care for patients, medical service providers often rely on advanced investigative tools that can help reveal hidden connections and patterns within the data. These tools can help providers gain valuable insights, which can lead to a more comprehensive understanding of patients’ needs and facilitate the delivery of high-quality care. Ontologies make it possible to search, extract, keep track of, and create data with a great degree of variety. The information must be shared across multiple channels and networks to address public health issues, but it may need to be more consistent with domain-specific language. These data sources are being used with ML in predictive analytics, precision medicine, and differential diagnosis. Ontologies may be used to store clinical data for ML and other types of computer research. Ontologies as a means of describing knowledge may be trustworthy, as they allow data to move freely between systems [105,106].

Phenotypic ontologies constitute a fundamental component of computational phenotype analysis. Using computational analysis to study phenotypes is a crucial component in comprehending the biological significance of genomic data within biological sciences. Phenotype ontologies have been devised across various fields and can be utilized to consolidate and scrutinize extensive interconnected datasets. The phenotype and trait ontology framework offers a systematic approach to defining phenotypes and their corresponding datasets, essential for developing techniques to integrate and analyze phenotype data [107]. Phenotype refers to any deviation from normal morphology and physiology in medical terminology. Over time, no large-scale, longitudinal data representations have been accomplished using phenotyping. The information gathering was time-consuming, partially finished, and provided generically. The utilization of deep phenotyping can provide new kinds of massive data, higher levels of specificity, and potential links between genetic variants and disease subtypes [108].

HPO is a highly comprehensive resource utilized for computational deep phenotyping and has become the de facto standard for deep phenotyping in the rare disease domain. It serves multiple purposes, such as enabling computable disease definitions and clinical abnormality descriptions, and facilitating genomic diagnostics. It is a hierarchical classification of standardized human pathological features that are used to define phenotypes [109]. The collaborative method of using AI, ML, DL, ontology, and HPO is the most effective technique for facilitating the interpretation of health information, and thereby improving understanding of the symptoms associated with long COVID.

In early 2022, Pfaff et al. [95] developed an ML approach using synchronized EHR data integrated into the safe N3C Data Enclave to identify potential patients with long COVID. Any person aged 18 years or older with a positive SARS-CoV-2 polymerase chain reaction (PCR) or antigen test was considered part of the study’s base population. The researchers used the three-site subset to generate three distinct machine learning models: (i) for all patients with COVID-19; (ii) for patients hospitalized with COVID-19; and (iii) for patients who had COVID-19 but were not hospitalized. Each model used the patient’s attendance at a long COVID clinic to indicate whether the patient was more likely to have long COVID. Researchers noticed that although individuals hospitalized for acute COVID-19 were primarily Black, the majority of long-term COVID clinic patients who were not hospitalized were female. The study’s findings revealed that the three models and long COVID manifestations had four common characteristics such as respiratory, non-respiratory, and pre-existing risk factors for increased severity of acute and chronic COVID symptoms, as well as signs that someone needed to go to the hospital.

Late in 2022, Reese et al. [109] created computer tools to assist physicians in comprehending how and why some individuals develop long COVID symptoms. This study highlights whether long COVID can be divided into well-defined and reproducible subtypes or whether heterogeneity is too significant to stratify. Because of the abovementioned factors, this is critically relevant for defining subcohorts in clinical research studies such as the NIH’s RECOVER program and finding viable treatments. They used a database of EHR data from 6469 patients who were diagnosed with long COVID after having a confirmed COVID-19 infection. HPO converts EHR data to phenotypic profiles. They applied a novel ML algorithm to cluster patients into six distinct groups, which they then characterized by analyzing substantial correlations between different COVID subtypes of symptoms, each revealing a distinctive long COVID subtype. Each cluster has different prior comorbidities and demographic variables, such as age, gender, and racial frequencies.

An article was published in the Journal of Natural Medicine [110] in early 2023 presenting a comprehensive analysis summary of the data-driven identification of subphenotypes associated with post-acute SARS-CoV-2 infection. According to that summary, the ML algorithm identified four reproducible clinical subphenotypes of long COVID. This software analyzes EHR data and aims to provide insights into long COVID by identifying common symptoms among individuals and classifying condition subtypes. Using ML to classify patients into groups based on patient–patient similarity scores and other demographic characteristics, six unique phenotypic clusters of long COVID patients, including clusters with distinct neuropsychiatric, pulmonary, and cardiovascular abnormalities, were identified. Cluster status was associated with a variety of pre-existing conditions and measures of disease severity. The researchers concluded that the tool serves as a basis for patient subgroup stratification, which could lead to further research and precision therapeutic care options. Follow-up research is needed to confirm these subphenotypes in patient populations from other geographical areas in the United States and other countries.

## 3. A Visionary Roadmap for Health Informatics Using Ontological-Based Concept Map

In order to assist healthcare professionals in attempting to manage the long-term effects of COVID-19, NICE, SIGN, and RCGP have teamed up. This collaboration has created a COVID-19 rapid guideline, which aims to assist healthcare professionals in navigating the challenges posed by this pandemic. In December 2020, a guideline for healthcare professionals regarding the management and care of individuals experiencing prolonged effects of COVID-19 was published. The scope of this provision pertains to the management of individuals exhibiting persistent clinical symptoms continuing beyond four weeks, which have arisen concomitantly or subsequently due to an infection compatible with the pathophysiological characteristics of COVID-19, which cannot be accounted for by other differential diagnoses. The guidelines provide a comprehensive definition of the effects of long COVID and provide evidence-based recommendations for the diagnosis and treatment of the disease. The aforementioned assertions are substantiated by robust evidence and a team of qualified professionals. Regular assessments of areas and the incorporation of new data and specialized expertise will be conducted. PASC clinics have implemented various leadership strategies, including primary and collaborative models involving internal medicine to infectious disease specialists. These strategies are aimed at addressing the PASC infection. The successful execution of an integrated referral system between PASC clinics, primary care providers, and core subspecialists is imperative, as it constitutes a pivotal component of a multidisciplinary approach [23,111].

A compendium of guideline recommendations that were published on 3 November 2022 has been collated, with a particular emphasis on primary care. The NICE website has the most contemporary data, and the present guidelines include a wide range of topics, including the following: (i) assisting people who have recently contracted COVID-19 and are still experiencing symptoms by identifying their identity; (ii) executing an initial assessment of individuals exhibiting symptoms persisting for more than four weeks to ascertain the existence of long COVID; (iii) providing specific investigations that are tailored to the individual’s long-term COVID-19 symptoms; (iv) examining the methods for planning care for people who are still having symptoms of COVID-19 or post-COVID-19 syndrome; (v) referring patients to multidisciplinary rehabilitation services for personalized management guidance and assistance; (vi) managing care for patients through recognizing the need for follow-up, monitoring, and discharge; and (vii) prioritizing consistency, perfect organization, and timely and liberal provision of care and services, which are extremely important [23].

**Semantic Network and Ontologies:** In 1896, Charles Peirce introduced his existential graphs, which were utilized to represent logical sentences in the form of diagrams consisting of nodes and links. The development of semantic networks was a result of node-and-link diagrams. Subsequently, other notations, such as conceptual graphs, were developed, although their syntax and semantics varied slightly. Despite their differences, all semantic network formalisms share a common goal of illustrating object groups’ hierarchical structure and interrelationships. A semantic network is a form of graph in which the nodes stand in for concepts and the connections between them are represented by the lines. They provide a methodical way to evaluate statements related to a particular topic [112,113]. Ontologies are recognized as advanced semantic systems that have been created over the last few decades to integrate essential domain concepts and their relationships as shared knowledge [114]. Semantic networks provide a foundation for structuring and connecting these concepts in a meaningful way.

**Concept Maps and Ontologies:** Concept maps, generated either manually or by using specialized software tools, depict information in a visual manner and emphasize the connections between different concepts or ideas in a specific domain. Semantic networks act as a valuable resource for concept map creation as they assist in the identification of concepts, determination of relationships, and structuring of the map, and in providing a starting point for customization. Concept maps play a significant role in the development and maintenance of ontologies by improving the organization, acquisition, conceptual clarity, consistency, and communication of knowledge. They facilitate effective representation and understanding of ontologies across different domains. Semantic networks and concept maps can be used to model ontological knowledge of a domain by representing the various entities and relationships between them [115,116].

**Figure 2:**
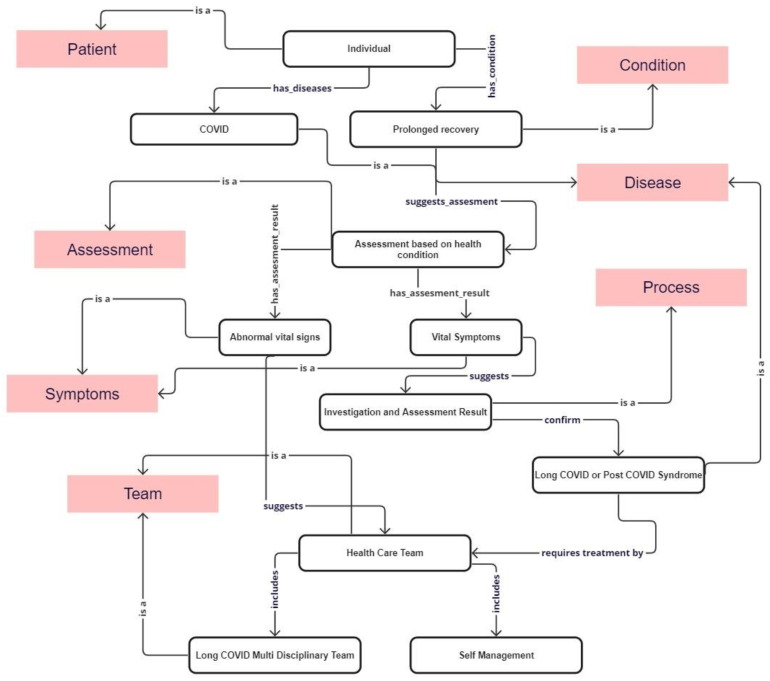
Part of the concept map for managing long COVID. This concept map version still needs to be completed, as the full version is currently undergoing a patent filing process.

**A Creation of a Concept Map for the Clinical Pathways of Long COVID Domain to Manage the Long-term Effects of COVID-19:** We were prompted by the NICE guidelines and used them as a conceptual foundation for developing a concept map for clinical pathways to address the challenges posed by long COVID (Figure 2), which have the potential for integration into a health informatics framework. The concept map was built by integrating different classes within the network to demonstrate the utilization of meta-relations as object properties for the long COVID domain. After identifying the primary domains, conducting a more in-depth analysis based on the fundamental requirements is necessary to extract the specific requirements for each domain.

We provided a comprehensive description of the classes (Table 1); mainly decision points involved in diagnosing and managing long COVID. The ontology models various concepts and captures the decision points, which are essential for making informed decisions regarding the management and diagnosis of long COVID.

The presented concept map at this stage represents an initial version focusing on standardized higher-level concepts rather than specific details in a medical ontology. This higher-level representation is a valuable tool for visualizing and comprehending the complex relationships within the concept map. In order to compare different long COVID concept maps, we plan to conduct a thorough evaluation of their structural and semantic similarities. This evaluation will utilize well-established techniques such as hierarchical correspondence, concept overlap metrics, and competency question alignment. The findings from this evaluation will guide an integration process, aiming to consolidate the most valuable components of each map into an optimized consolidated version.

## 4. Discussion on Future Direction

We provided an ontological-based concept map due to its effectiveness in organizing and representing complex information clearly and concisely. Ontologies are often used in biological and biomedical research due to their combination of features, including standard identifiers for classes and relations, standardized nomenclature, metadata, and machine-readable axioms and definitions. The combination of these features to facilitate integrative and intelligent analysis and interpretation of biomedical data has the potential to be transformational. These properties allow applications that ease data integration, data access, and data analysis [117,118]. To specifically collect and manage information related to long COVID, developing and implementing strategies and processes for identifying the most pertinent conceptual dimensions and organizing them appropriately is necessary. This requires collecting and managing medical and clinical data from numerous sources. An ontology network is a collection of ontologies derived from different knowledge domains and connected through meta-relationships. Developing an ontology network to monitor the long-term effects of COVID-19 is one suggested strategy [119]. Ontologies are useful for studying and managing long COVID. Using these ontologies, researchers can systematically organize and analyze data about long COVID. As a result, it is possible to identify potential risk factors, formulate targeted treatments, and disseminate findings throughout the scientific community. Incorporating ontologies in long COVID research facilitates a comprehensive and standardized methodology for investigating and addressing this emerging health condition. Concept maps and semantic networks provide adaptable and visible representations of information. By defining ideas and connections, both concept maps and semantic networks may be used as building blocks for ontologies.

Recently, it was shown that the collaborative approach is the most successful strategy for generating, maintaining, and reusing ontologies. This paper explores the immense potential of technological advancements in addressing the long-term effects of managing COVID-19. Specifically, it focuses on utilizing concept mapping as a valuable tool within the domain of long COVID. By integrating various distinct classes into a single ontological concept map, this approach creates a comprehensive network of knowledge. In contrast to the current fragmented approach, where physicians and healthcare providers offer individual services, this all-encompassing method ensures that patient records remain the primary and accurate source of information in a single place.

The innovative approach presented in this paper highlights the effective utilization of concept mapping in managing long COVID. The integrated ontological concept map holds great promise for developing a complete solution with maximum features. This strategy significantly lessens the burden experienced by medical professionals, from primary care providers to multidisciplinary team members, by providing a comprehensive view of clinical interpretation and reducing the challenges posed by patient ignorance, language barriers, missing medical records, and other obstacles.

Health informatics is a multidisciplinary domain that integrates healthcare, information technology, and data management to enhance healthcare outcomes, optimize patient care, and streamline healthcare processes. Using the concept map method, programmers can create software and applications that can be integrated into the domains of health informatics as a variable risk factor subdomain. These applications serve as E-human services, forming an emotionally supportive network to enhance healthcare service delivery and promote patients’ well-being. Reducing the amount of time spent in transit to and from medical facilities promotes prompt access to specialized social insurance administrations, which vary according to the patient’s or case’s previous medical history. It provides the manager of human services with a rapid and up-to-date comprehension of health information, so it cuts down on paperwork and conventions and increases the ease with which the data may be used more effectively and reliably by therapeutic foundations.

Altogether, this research presents a constructive and progressive strategy for tackling the complexities of long COVID management. By leveraging the power of concept mapping and an integrated knowledge ontology, this innovative strategy aims to provide medical professionals with a valuable resource that enhances patient care, streamlines processes, and promotes effective collaboration across the healthcare ecosystem.

## 5. Conclusions

The global public health community has faced an unprecedented challenge with COVID-19, and long COVID continues to represent a serious threat to the public’s health. Understanding long COVID’s significance is crucial for effective management, exploring reversibility, and identifying prevention and intervention opportunities. The research question explored the management gaps of long COVID, investigating how addressing these gaps and creating a standardized higher-level concept map could improve a structured approach to managing long COVID, in line with our hypotheses. By utilizing the potential of this strategy, we can reduce the burdens on patients, caregivers, and physicians while enhancing health outcomes by applying personalized, well-informed care plans. The continuous evolution of health information technology offers a remarkable chance to address the challenges of long COVID and strengthen public health readiness for upcoming infectious disease outbreaks. This review provides evidence-based recommendations for addressing long-term COVID management, significantly contributing to the growing field of knowledge. It emphasizes the importance of continuous research and implementation efforts to protect the well-being of individuals affected by long COVID and other emerging health threats.

## Figures and Tables

**Table 1:** Class used in decision points.

Class	Definition
Patient	Individual with a specific disease or condition
Condition	Conditions refer to the presence of a specific health condition over time
Assessment	Health assessment to evaluate the health of the patient
Process	Any medical investigation that requires the use of medical equipment or a laboratory
Symptoms	Defines how a person feels about specific conditions
Disease	Person experiencing physical or mental problems indicating a disease or condition
Healthcare team	Defines the expert care team collaborating to provide treatment

## Data Availability

The datasets generated and/or analyzed during the current study can be obtained from the corresponding author upon submitting a reasonable request.
